# Comparative classification of species and the study of pathway evolution based on the alignment of metabolic pathways

**DOI:** 10.1186/1471-2105-11-S1-S38

**Published:** 2010-01-18

**Authors:** Adi Mano, Tamir Tuller, Oded Béjà, Ron Y Pinter

**Affiliations:** 1 Dept. of Computer Science, Technion - Israel Institute of Technology, Haifa 32000, Israel; 2 School of Computer Science, Tel Aviv University, Ramat Aviv 69978, Israel; 3 Department of Molecular Microbiology and Biotechnology, Tel Aviv University, Ramat Aviv 69978, Israel; 4 Dept. of Biology, Technion - Israel Institute of Technology, Haifa 32000, Israel; 5 Google Haifa, MATAM 30, Haifa 31905, Israel; 6 Faculty of Mathematics and Computer Science, Weizmann Institute of Science, Rehovot 76100, Israel

## Abstract

**Background:**

Pathways provide topical descriptions of cellular circuitry. Comparing analogous pathways reveals intricate insights into individual functional differences among species. While previous works in the field performed genomic comparisons and evolutionary studies that were based on specific genes or proteins, whole genomic sequence, or even single pathways, none of them described a genomic system level comparative analysis of metabolic pathways. In order to properly implement such an analysis one should overcome two specific challenges: how to combine the effect of many pathways under a unified framework and how to appropriately analyze co-evolution of pathways.

Here we present a computational approach for solving these two challenges. First, we describe a comprehensive, scalable, information theory based computational pipeline that calculates pathway alignment information and then compiles it in a novel manner that allows further analysis. This approach can be used for building phylogenies and for pointing out specific differences that can then be analyzed in depth. Second, we describe a new approach for comparing the evolution of metabolic pathways. This approach can be used for detecting co-evolutionary relationships between metabolic pathways.

**Results:**

We demonstrate the advantages of our approach by applying our pipeline to data from the MetaCyc repository (which includes a total of 205 organisms and 660 metabolic pathways). Our analysis revealed several surprising biological observations. For example, we show that the different habitats in which Archaea organisms reside are reflected by a pathway based phylogeny. In addition, we discover two striking clusters of metabolic pathways, each cluster includes pathways that have very similar evolution.

**Conclusion:**

We demonstrate that distance measures that are based on the topology and the content of metabolic networks are useful for studying evolution and co-evolution.

## Background

The increasing availability of pathway information enables the comparative analyses of organisms at the functional level. As the basis for such studies we propose to use *pathway alignment*, an effective technique that provides both a similarity score as well as a clear indication of specific differences between pathways. The score can be used as yet another type of data with which to build dissimilarity-based phylogenies, but - in addition - the alignment details allow in-depth analysis of the evolutionary changes that various pathways underwent. Thus, our method allows a systematic examination of evolutionary relationships among species using their functional traits, based on a description of their entire metabolic processes rather than an individual element as in conventional analysis. In addition, such studies allow us both to further improve pathway alignment techniques by finding more faithful similarity scores, as well as to study co-evolution of metabolic pathways.

Specifically, there is currently a significant amount of metabolic pathways data residing in public and proprietary databases such as KEGG [1], MetaCyc [2], iPath [3] and BioCarta [4]. The various databases have mostly overlapping data; however, the pathways of many organisms appear only in some of the databases, or appear in all or some of them but with different constituent enzymes and morphology. Nevertheless, this data is rich enough to be used in order to determine similarity in a large set of species and pathways.

A single analogous pathway can be used as the basis for generating a similarity matrix between a set of species (*i.e. *the similarity score that is obtained when comparing this pathway in each pair of organisms is regarded as the similarity between them), and then build a phylogenetic tree that reflects the evolutionary history based on this pathway alone. One pathway, however, does not tell the whole story. The first algorithm that we propose here looks at all the known pathways in a set of organisms and combines their similarity scores in a systematic fashion to produce a more comprehensive picture; an entropy-like weight determines the information contents of each individual pathway when computing the combined similarity score between organisms.

Furthermore, pathways interact functionally with each other; these interactions can be reflected in their evolution. Our second algorithm is a novel approach for studying the co-evolution of metabolic pathways. Our method detects pathways whose evolution is correlative both in terms of changes in their topology and in terms of changes in their enzymatic content.

The application of our algorithms enabled us to draw some specific conclusions about the usability of pathway information. Moreover, we were able to make observations about the evolutionary relationships between certain families of organisms and shed light on their proper classification. For example, we were able to resolve evolutionary relationships among different archaeal species based on their metabolic pathways. Furthermore, unique properties common to both thermophilic *Crenarchaea *and *Euryarchaea *were detected. Additionally, we used our approach to study co-evolution of metabolic pathways. We discovered that the analyzed pathways can be clustered into two groups according to their evolution. This result may imply that there are strong functional relations between pathways that are part of the same cluster.

### Previous work

Both the algorithm for inferring phylogeny from metabolic pathways and the algorithm for studying co-evolution of metabolic pathways are based on the alignment of metabolic pathways [5]. There has been considerable work on the alignment of pathways and networks [5-7] and most methods provide some kind of a similarity score; we refer the reader to [8] for a recent review. Dandekar *et al. *[9] presented one of the earliest efforts introducing comparative analysis of metabolic pathways, combining three methods of comparing biochemical pathways: analysis and comparison of biochemical data, analysis based on elementary modes, and comparative genome analysis. Among other results, it showed high plasticity in the glycolysis pathway. Clemente *et al. *[10] used metabolic pathway comparison to show that vital biological processes in a group of related species are usually expressed by a number of highly conserved reactions. Furthermore, they show that it is unlikely for a group of reactions to be completely missing form one of the organisms in a set of similar species.

There has also been growing interest in the reconstruction of phylogenetic trees from the abovementioned comparison results [11-15] in recent years. Heymans and Singh [14] presented a technique for the phylogenetic analysis of metabolic pathways based on the topology of the underlying graphs. They defined a distance measure between graphs using the similarity between nodes of the graphs (and some consideration of the topological relationship between them). This distance measure was applied to the enzyme-enzyme relational graphs derived from metabolic pathways and the resulting distance matrix was used to obtain a phylogenetic tree. In a later work by Clemente *et al. *[16], a software tool that uses metabolic pathway comparison to create phylogenetic trees is presented; the comparison method used here is pseudo-alignment, *i.e.*, mapping each reaction in one pathway to another reaction or a group of reactions in the other. This tool, however, covers only a limited number of organisms and pathways for which a phylogenetic tree can be built. Hong *et al. *[17] presented a method for constructing a phylogenetic tree based on metabolic data deduced from genomic sequences. This method accounts for horizontal gene transfer and specific gene loss by comparing whole metabolic subpathways, and allows evaluation of evolutionary relatedness and changes in metabolic pathways.

Another work in this area is by Chor and Tuller [18]. They create phylogenetic trees based on distances between metabolic networks of different species. The distance measure they use is based on the relative description length (RDL) of the networks, *i.e. *the number of bits needed to describe one network given the other. This method is more efficient when dealing with large metabolic networks than most other known comparison methods.

Still, to the best of our knowledge, to date (except for the MetaPathwayHunter, MPH, alignment algorithm of [5] on which our paper is based) no one has used a score that reflects both the pathway structure as well as the identity of the enzymes in such studies and no systematic handling of all pathways in many organisms has been offered.

Finally, previous works showed that proteins with similar functionality [2,19,20] that physically interact [21], or that are close to each other in a metabolic network [22,23], tend to have similar evolution. However, to the best of our knowledge, no previous work analyzed co-evolution of metabolic pathways by a measure that considers both the enzymes in the pathways and its topology.

Here we perform for the first time a complete computational comparison of organisms based on all the available metabolic pathways that describe their function, taking into account both enzyme contents as well as pathway topology, and accounting for differences in functionality that is reflected in missing pathways. Furthermore, we deduce the co-evolution of pathways in different organisms based on the same data. Our results reveal a few novel and biologically significant observations.

## Methods

### Pathway alignment and data organization

Most pathway databases and repositories (such as KEGG [1], MetaCyc [2], and BioCarta [4] provide an instance of each pathway for each of the organisms in which this pathway occurs. Instances describing the same cellular process could be different, of course, but they are considered analogous. Thus, differences between two organisms that are specific to a given pathway can be found by using a pathway alignment engine such as MetaPathwayHunter (MPH) [5], to align the two instances against each other, producing (the not necessarily equal) two similarity scores. When looking at a set of *n *organisms, we can arrange the results of an all-against-all comparison of the organisms for a specific pathway in an *n *× *n *matrix ***a***, where *a*_*ij *_is the similarity score of aligning the pathway as it appears in organism *i *with its analogous occurrence in organism *j*.

**Figure 1:**
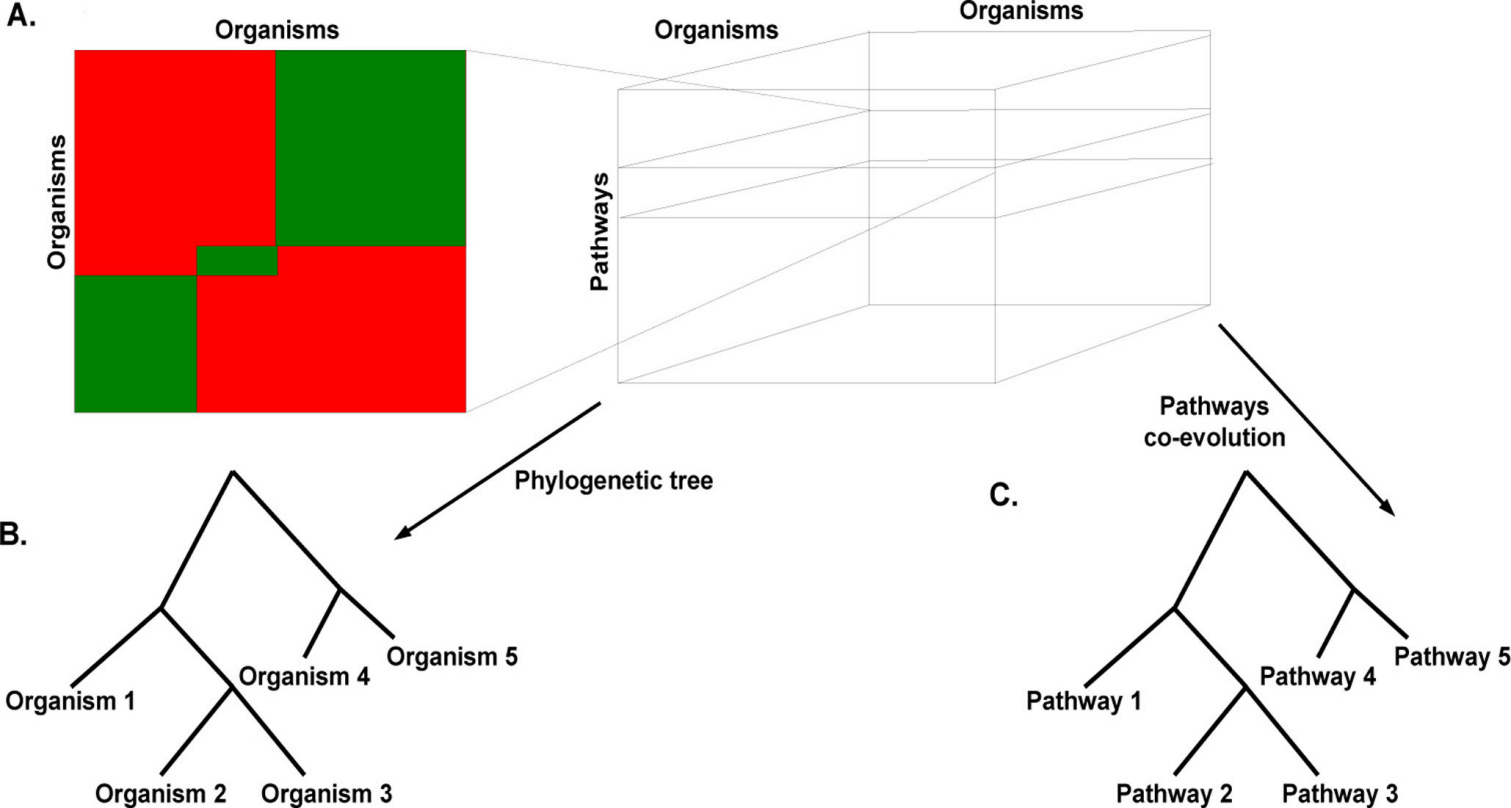
**The "pages" matrix and its uses**. (A) A "page" summarizes the alignment results for one pathway which actually describes the evolution of a pathway; Many such pages, stacked on top of each other, create an organisms*organisms*pathways 3D matrix. The 3D matrix can be used for building phylogenetic trees (B) while comparison of pages can be used to study co-evolution of pathways (C).

Each such 2 dimensional array is called a *page *(Figure 1A), and in itself it constitutes a similarity matrix that can be used for building a phylogeny. A single pathway, however, is not a good enough basis for deriving meaningful phylogenies (and for calibrating the similarity score). Rather, we are interested in a global picture based on all the available pathways. Thus, we create a page for each of the *m *pathways in the database; then, the resulting pages are organized in an *n *× *n *× *m *3 dimensional (3D) array, ***A***, where each page is indexed by the corresponding pathway (Figure 1B); now *A*_*ijk *_is the similarity score for comparing pathway *k *as it appears in organisms *i *and *j*. Note that since some pathways do not exist for some of the organisms (or are missing from the database), the sizes of the pages might not be commensurate with each other; to accommodate for this phenomenon and to obtain a regular *n *× *n *× *m *3D array we stretch the pages to be the same size, denoting missing entries appropriately (see more on this below).

### Interpreting and weighting the data

Analyzing these data in order to obtain insights about the evolutionary history of the organisms was performed as follows: We collapse the 3D array into a 2-dimensional (dis)similarity matrix by combining the *m *entries in each column into one scalar entry; this matrix would serve us as a distance matrix in order to build a phylogenetic tree using known algorithms. The first step in the process of combining a column's entries is setting a threshold that describes scores as relevant: all similarity scores below this threshold (assuming all similarity scores, as produced by MPH, are lower than or equal to 0 and the higher the score - the better the similarity) will not contribute to the values in the similarity matrix, and all similarity scores above it will contribute to them equally. Using this method - on one hand - causes some loss of information, but - on the other hand - enables combining the data in an effective manner while also handling the issue of missing entries that will be discussed in more detail later in this section. For each page we create a *Threshold Graph *(TG) in the following manner: define a node *v *∈ *V *for each organism; two such nodes are connected by a directed edge *e *∈ *E *if their similarity score is higher than the given threshold. Using (1) we calculate *w*, an assessed weight for each TG:(1)

This weight, inspired by (but not identical to) Shannon's entropy [24], reflects the amount of information in the page. This formula gives scores ≥0 to each graph, based on the degrees of all its nodes. A graph will have *w *= 0 if the degrees of all nodes in the graph are 0, since the  part of the formula will be 0 for each node.

Alternatively, w = 0 if all node degrees are |V|, in which case the  will be equal to 0 for all nodes. In the first case, the organisms are all different from one another and there is nothing to learn about their similarity. In the latter, each organism is "very similar" to all the other organisms, which can teach us nothing new about the whole group. Each edge in the TG is given a weight according to this formula. The 3D array is modified by replacing *A*_*ijk *_by 0 if nodes *i *and *j *are not joined by an edge in the TG corresponding to pathway *k*, and by the calculated weight *w *if an edge exists between organisms *i *and *j *in the TG. In fact, each page is now the adjacency matrix of the weighted TG.

This technique also solves the problem of missing entries, as follows: for the cases where a pathway exists in one of the organisms but does not in the other, we decided never to connect the two organisms with an edge in the TG, and as a result the corresponding entry in the array will always be 0. To accommodate the cases where the pathway is missing in both organisms, we added a new global, Boolean parameter *b*: If *b *is true then each such two organisms will be joined by an edge, otherwise such edges will not be formed. Intuitively, if the confidence in the data is high then *b *should be assigned true, since in this case the absence of a pathway in both organisms tells us that they are similar in their lacking. If our confidence in the data is low, *b *should be assigned false since in this case the pathway could be missing due to lack of data, and this might not indicate a real (dis)similarity.

Naturally, using a threshold in forming the TG resulted in loss of information. However, we found that the advantage of being able to include missing entries is far more significant to our results than the information lost by not using the actual alignment score. Furthermore, by adjusting the threshold value, we can make sure that our results are as precise as possible. After dealing with this issue we can safely add all the similarity scores in each vector along the pathway axis and thus create a 2-dimensional similarity matrix.

### Building phylogenetic trees

The resulting 2D matrix is turned into a dissimilarity matrix so that it can be used as input to an algorithm for building phylogenetic trees, *e.g. *the neighbor-joining algorithm [25], as embodied in *e.g. *the Phylip [26] package, as follows: each element in the matrix is subtracted from the maximum element in it; recall that this element must appear along the main diagonal since each organism obtains a perfect similarity score when aligned with itself.

Finally, it is possible to identify the contribution of specific pathways to the resulting phylogeny: One can trace differences among phylogenetic trees back to the pathways that cause them in order to understand their biological reason; furthermore, the alignments produced by the MPH algorithm can be used to further elucidate these differences. Moreover, we can study the pathways whose TGs got the highest weights to see if they induce an interesting classification of the organisms into groups.

### The computational pipeline for inferring a phylogenetic tree based on metabolic pathways

The pseudo-code for the computational pipeline is given here:

**Input**:   n: number of organisms

m: number of pathways

P: matrix n*m. Entry P_ij _is the instance of pathway j in organism i

d: deletion score for MPH

t: threshold

b: Boolean parameter to determine whether a pathway missing in two organisms is considered over (*b *= true) or under (*b *= false) the threshold

**Output**: tree: a phylogenetic tree

**for **k := 1..m **do**

   **for **i, j := 1..n **do**

      **if **exists P_ik _and exists P_jk _**then**

         **if **size of P_ik _< P_jk _**then **A_ijk _:= MPH(P_ik_, P_jk_, d);

         **else **A_ijk _:= MPH(P_jk_, P_ik_, d);

      **elsif **not exists P_ik _and not exists P_jk _**then **A_ijk _:= *both missing*;

      **else **A_*ijk *_:= *one missing*;

   build graph TG(V, E) s. t.

      V := {1..n};

      E := {(i, j) | A_ijk _≥ t}; //*only numeric values*

      **if **b **then **E := E ∪ {(i, j) | A_ijk _= *both missing*};

   ;

   **for **i, j := 1..n **do**

      **if **(i, j) ∈ E **then **A_ijk _:= *w*(*TG*(*V*, *E*));

      **else **A_ijk _:= 0;

**for **i, j := 1..n **do**

   SimMat_ij _:= ;

DisSimMat := SimMat_11_*1_(n, n) _- SimMat;

tree := NeighbourJoining(DisSimMat);

The time complexity of this algorithm is *O(mn*^2^), which is dominated by the time needed to read the entries of the input array, assuming that *m *>>*n*.

### Data sources

Our algorithm was applied to data that was obtained from the MetaCyc repository [2]. We chose this database since it is very rich in data and it is organized in a way that best suits our requirements, *i.e.*, it contains one variation of each pathway for all the organisms for which it is known. Initially we used over 660 metabolic pathways for the 205 organisms that were retrieved from this database (the entire data available at the time of the assay, September 2007). This presented a significant computational challenge, namely making over 26 million individual pathway alignments. Fortunately, this problem is "embarrassingly parallel", *i.e. *each alignment can be run separately, and thus we were able to solve it using a computational Grid facility.

### Studying co-evolution of metabolic pathways

For comparing the evolution of different metabolic pathways the following steps were performed. First, a distance matrix (*i.e. *a page, see Figure 1) was computed for each pathway (as before). Next, all the pages were compared to each other, one pair at a time. To this end, we considered both the fact that a pathway may appear only in part of the organisms and that in cases that a pathway appears in a pair of organisms its structure in the two organisms may be different (see the previous subsections). Thus, the distance measure between a pair of matrices includes two components: one is the generalized Hamming distance that 'captures' similar/non-similar appearances in the same organism, *H*(*p*_1_, *p*_2_). This can be further explained as follows: Let *d*_1 _denote the value of an entry in the distance matrix corresponding to a case where a pathway does not appear in the two organisms; let *d*_2 _denote the value of an entry in the distance matrix corresponding to a case where a pathway appears in the two organisms; let *d*_3 _denote the value of an entry in the distance matrix corresponding to a case where a pathway appears in the first organism but not in the second one; and let *d*_4 _denote the value of an entry in the distance matrix corresponding to a case where a pathway appears in the second organism but not in the first one. Let *D*(*x*, *y*) denote the contribution to the generalized Hamming distance due to value *x*in a certain entry in the page of one pathway and *y *in the same entry in the page of the second pathway. We used ∀_*x*_*D*(*x*, *x*) = 0;

*D*(*d*_3_, *d*_4_) = *D*(*d*_4_, *d*_3_) = 2, *D*(*d*_3_, *d*_1_) = *D*(*d*_4_, *d*_1_) = *D*(*d*_3_, *d*_2_) = *D*(*d*_4_, *d*_2_) = 1, and *D*(*d*_1_, *d*_3_) = *D*(*d*_1_, *d*_4_) = *D*(*d*_2_, *d*_3_) = *D*(*d*_2_, *d*_4_) = 1.

The final score was normalized by dividing it by the number of entries in a page.

The second component, *L*(*p*_1_, *p*_2_), considers only entries where both pathways appear in both organisms and it is the *L*_1 _distance between the vectors that are composed of these entries in the two organisms. The final distance is a weighted average of the two distances, computed as in (2). As the first component of the score reflected a rougher distance measure we used *W*_*p *_= 100; however, the result was robust to small changes in *W*_*p*_.(2)

Based on this measure we generated a distance matrix between pathways that can be further analyzed as described in the next section (for example by clustering analysis).

## Results and discussion

### Analysis of the entire dataset of MetaCyc

The version of MetaCyc that was used in this work includes 660 pathways and 205 organisms. Using a computational grid comprising 250 nodes we were able to analyze the entire dataset, *i.e. *to perform more than 26 million individual pathway alignments in just a few days.

Evolutionary trees based on the entire dataset were very noisy. The main reason for this result is the fact that the data in most of the pathways/organisms are very partial. For example, the mean size (number of enzymes) of 200 of the pathways over the organisms that appear in the dataset is less than 3. On the other hand, the size of the pathway with the top mean size is 53. Thus, as specific biological examples (as described in the next subsections) we chose less biased subsets of organisms and pathways.

### An example: a phylogenetic tree of Archaea based on metabolic pathways

As a specific biological example we performed a deep biological analysis of all the Archaea that appear in MetaCyc based on their metabolic pathways. We chose this dataset for two main reasons. First, this dataset is less biased since the size of the metabolic network of all Archaea in MetaCyc is relatively similar (up to 56% difference; for example, in Eukaryotes the maximal difference was 430% and in Bacteria it was much larger, 720%). Second, as the Archaea live in extreme and diverse environments it is interesting to compare the changes in their metabolic networks to their phylogeny.

**Figure 2:**
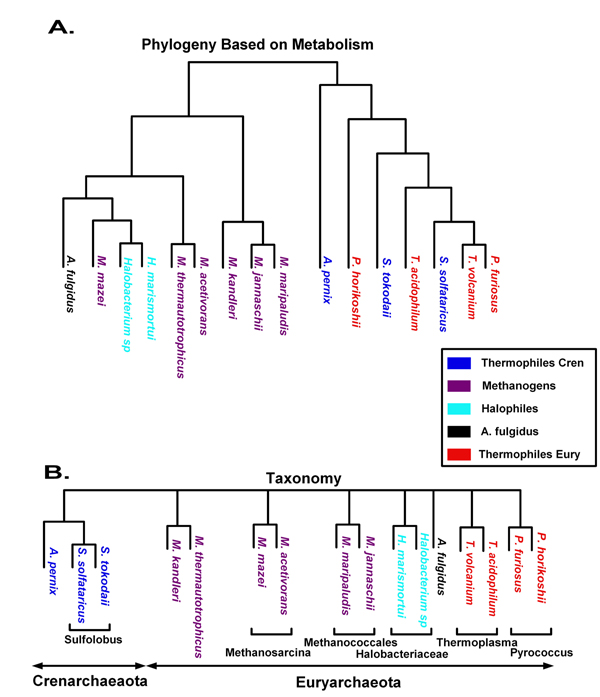
**Archaea taxonomical trees**. (A) A taxonomical tree for the analyzed Archaea as downloaded from the NCBI website. (B) A tree constructed for the Archaea by our algorithm using deletion score = -2, threshold = -5, b = true.

A phylogenetic tree that was produced by running the algorithm is displayed in Figure 2A and was compared to the NCBI reference tree (displayed in Figure 2B). Note that even though the two trees are differently structured, the basic topology is very similar, *i.e. *the resulting tree is well separated into Methanogens (*Methanococcus maripaludis*, *Methanococcus jannaschii*, *Methanopyrus kandleri*, *Methanosarcina mazei*, *Methanosarcina acetivorans*, and *Methanothermobacter thermautotrophicus*), Halophiles, and Thermophiles and *Archaeoglobus fulgidus *is farthest from the rest of the organisms.

The differences between these two trees are interesting and usually can be explained: The Hyperthermophilic *A. fulgidus *also falls close to the Methanogens. This can be explained by the fact that *A. fulgidus *represents an evolutionary transitional type of organism among the *Archaea*: it is a hyperthermophile and yet is known to be related to Methanogens [27]. For example, *A. fulgidus *contains acetyl-CoA decarbonylase/synthase (ACDS), a multienzyme complex catalyzing the reversible cleavage and synthesis of acetyl-CoA in methanogens [28]. *A. fulgidus *is an example of an organism that contains a functional ACDS complex and is not a methanogen. Furthermore, *Thermophilic Archaea *are clustered together (with representatives from both *Thermophilic Crenarchaea *and *Thermophilic Euryarchaea*). This implies the existence of unique pathways that are found only in thermophiles. Anaerobic respiration, TCA cycle, and tryptophan biosynthesis pathways in MetaCyc were found to include specific variations unique to thermophiles while chorismate biosynthesis, formaldehyde assimilation, threonine biosynthesis, and valine biosynthesis pathways were missing from thermophiles in the MetaCyc dataset. For example, in the chorismate pathway it is known that many *Archaea *do have a distinct shikimate kinase [29]. In addition, a new pathway (using a ribulose monophosphate instead of fixating formaldehyde) for the generation of pentoses needed for the chorismate pathway was recently found in thermophilic *Archaea *[30].

Another interesting discrepancy between the two trees is the clustering of *Methanosarcina mazei *with *Halobacteriaceae *in the tree that is based on metabolism. This result can be explained by the fact that *Methanosarcina mazei *is a freshwater organism that can adapt to grow at elevated salinities [31]. Thus, its metabolism resembles the metabolism of *Halobacteriaceae *that are found in water saturated or nearly saturated with salt.

Still, there are some unresolved discrepancies between the two trees. One unresolved issue is the intermix of the *Thermophilic Crenarchaea *and the *Thermophilic Euryarchaea *in the thermophilic cluster. Specifically, why are the *Thermophilic Crenarchaea *divided in the tree shown in Figure 2A? Why is *Pyrococcus horikoshii *linked to them whereas the crenarchaeote *Sofolobus solfataricus *is grouped with the euryarchaeotes *Pyrococcus furiosus*, *Thermoplasma acidophilum*, and *Thermoplasma volcanium*? This may be either a result of a curation error in the MetaCyc database or it may suggest that the metabolism of *Thermophilic Crenarchaea *and the *Thermophilic Euryarchaea *is not significantly different. We encourage an additional future research on this issue.

### Co-evolution of metabolic pathways

**Figure 3:**
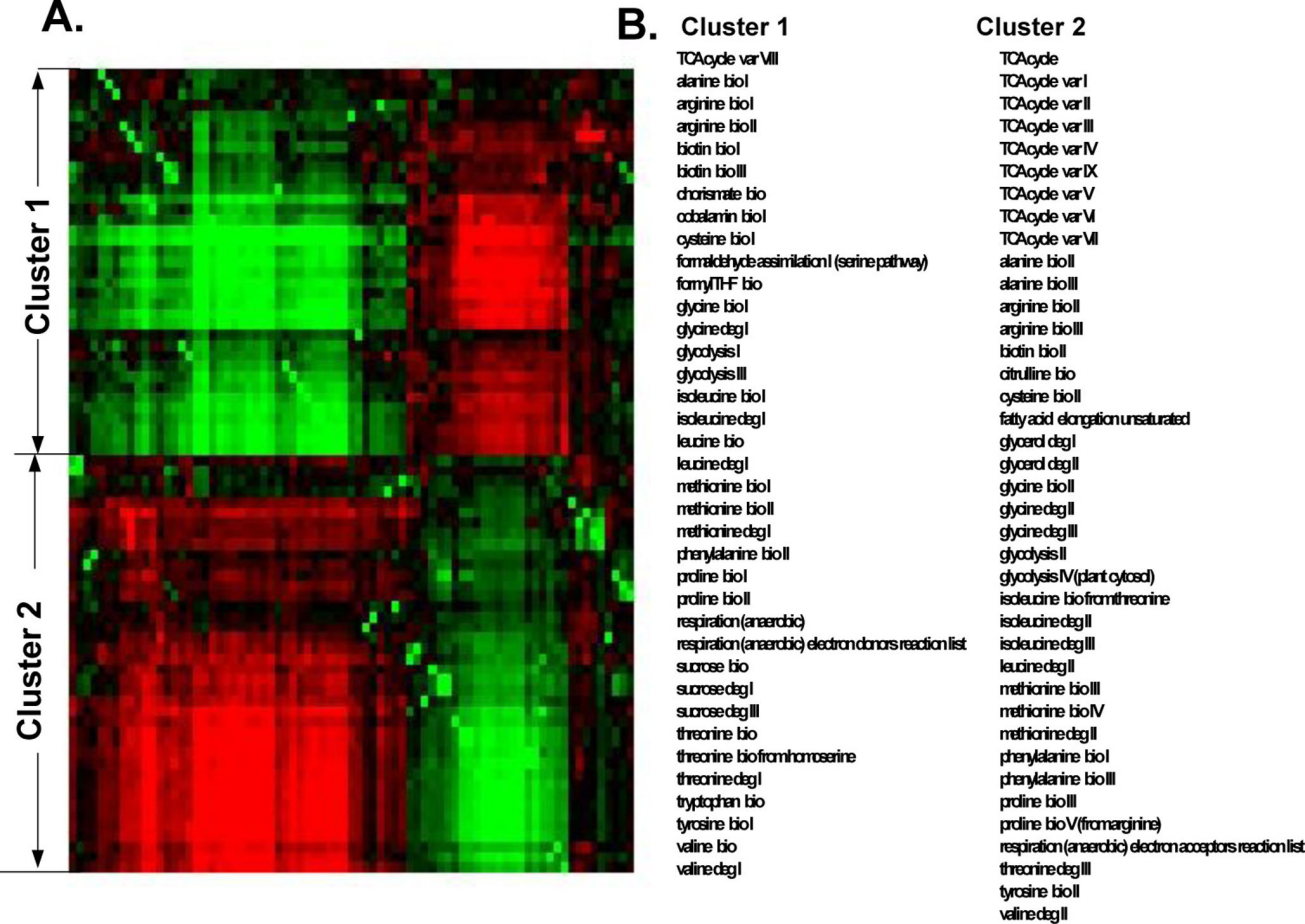
**Metabolic pathways clustering**. Clustering of metabolic pathways according to their evolution. (A) Clustering analysis reveals two clusters of pathways. Each cluster includes pathways with very similar evolution. Green indicates similarity whereas red - dissimilarity. (B) The list of pathways in each cluster; bio is an abbreviation of biosynthesis, deg is an abbreviation of degradation and is an abbreviation of variation.

We examined our approach for detecting co-evolution of pathways on a subset of the available pathways that are known to be shared by many organisms such as amino acids biosynthesis and degradation, glycolysis, TCA cycle, and more (a total of 77 pathways; see Figure 3B). These pathways have instances in most of the 205 organisms we were considering, which enabled a significant outcome. We generated a distance matrix between pathways as described in the previous section and used the Click algorithm [32] for clustering the pathways according to their corresponding rows in this distance matrix. Based on some probabilistic assumptions on the input, Click finds both the number of clusters and the partitioning of elements into clusters [32].

As can be seen (Figure 3A), two very striking clusters were observed. Each cluster includes metabolic pathways whose pages are very similar to each other and are non similar to pages in the second cluster (Figure 3). Specifically, pathways from the same clusters tend to co-occur in the *same *organisms and pathways from different clusters tend to occur in *different *organisms. The lists of the pathways in each cluster appear in Figure 3B. This result suggests that there is a very strong co-evolution of pathways: even though there are a few variants in each metabolic pathway that can theoretically correspond to a large number of clusters, only two clusters were observed. It encourages future biological studies about the functional constraints that shaped the observed co-evolutionary relations.

## Conclusion

In this work we describe a new approach for comparing the metabolism of organisms and for studying co-evolution of metabolic pathways. We show that by adopting this approach one can gain new interesting biological conclusions about the evolution of metabolism in sets of organisms.

Naturally, the study presented in this work can be generalized in various ways. First, when the data in the various pathway databases will be more reliable and robust, we shall be able to use our tool to construct a complete phylogenetic tree based on all the pathways and organisms in such a database (the same input as in Figure 1). By using different parameters (deletion score, the similarity score threshold, and the *b *parameter), different trees would be created. These trees could then be compared to reference trees using *e.g. *the Robinson-Foulds measure, and that way to determine the best values for the parameters by picking those yielding the best match. Furthermore, such a process could enable comparative studies among the pathway databases themselves; currently, they are incomparable in several ways (*e.g. *the granularity of the pathways, the coverage of species, and more), making such a study premature.

Another idea for future research is to consider an alternative method for reconstructing the phylogenetic trees: it would be to use an iterative process in which the pages of our 3D array are used - one at a time - to refine the constructed tree. One could sort the pages by their specificity, and at each one of the iterations, the next page would be used in order to refine the distribution of the organisms, and thus create a new level for the tree. This way one will be able to distinguish among pathways that are common to all forms of life, those that delineate kingdoms and families, all the way to those that constitute the detailed separation into species and even strains. Still, such an approach might present problems in finding the right order for selecting the pathways, and the method presented in this paper - which considers all the pathways together - could well prove advantageous.

Finally, we described here a new method for studying co-evolution of metabolic pathways. Our approach considers both the set of enzymes in a pathway and the topology of the pathway. In this work, we demonstrated this method by analyzing the evolution of 77 reliable metabolic pathways over 205 organisms. When reliable data about additional metabolic pathways will be available it will be interesting to use our approach for analyzing a larger set of metabolic pathways. It will be interesting to use such an analysis for comparing the evolution of entire pathways to the evolution of single genes (enzymes) that are related to the pathways. Additionally, a similar approach can be used for analyzing other cellular biological sub-networks (*e.g. *the co-evolution of complexes in protein-protein interaction networks).

## Competing interests

The authors declare that they have no competing interests.

## Authors' contributions

AM, RYP, OB and TT participated in the design of the study. AM and TT analyzed the data. AM, RYP, OB and TT wrote the paper. All the authors approved the final manuscript.
